# Direct randomized evidence for systemic targeted therapies in prurigo nodularis: a systematic review and meta-analysis

**DOI:** 10.3389/fmed.2026.1942040

**Published:** 2026-10-15

**Authors:** Yue Yu, Siqi Li, Weiwen Chen, Xinsheng Chen

**Affiliations:** 1 The Second School of Clinical Medicine, Guangzhou University of Chinese Medicine, Guangzhou, Guangdong, China; 2 Department of Dermatology, The Second Affiliated Hospital of Guangzhou University of Chinese Medicine (Guangdong Provincial Hospital of Chinese Medicine), Guangzhou, Guangdong, China

**Keywords:** prurigo nodularis, pruritus, randomized controlled trial, safety, sleep response, systemic targeted therapy

## Abstract

**Background:**

Systemic targeted therapies are reshaping treatment for prurigo nodularis (PN), but the timing of clinically meaningful benefits and the strength of supporting evidence remain unclear. We synthesized direct placebo-controlled evidence by outcome and follow-up time, distinguishing replicated phase 3 evidence from preliminary signals.

**Methods:**

PubMed, Embase, Web of Science, CENTRAL, and ClinicalTrials.gov were searched from inception to June 30, 2026, for randomized controlled trials of systemic targeted therapies for PN. Compatible outcomes were pooled separately at prespecified time points using random-effects models. The primary outcome was a ≥ 4-point reduction in weekly average Worst Itch or Peak Pruritus Numerical Rating Scale scores. Secondary outcomes included Investigator's Global Assessment (IGA) success, dual response, sleep, quality of life, psychological outcomes, and safety. Risk of bias and certainty were assessed using RoB 2 and GRADE.

**Results:**

Eleven trials involving 1,623 participants evaluated six therapies. Itch response favored active treatment at weeks 4 (RR 4.76, 95% CI 3.01–7.55), 12 (2.67, 1.43–5.00), 16 (3.32, 2.57–4.29), and 24 (3.09, 2.18–4.38), whereas the week 8 estimate was imprecise (1.73, 0.94–3.21). IGA success was supported from week 8 onward. Nemolizumab improved sleep response by week 4, and two phase 3 dupilumab trials showed a week 24 dual-response benefit (RR 3.98, 2.31–6.85). Treatment-emergent adverse events were modestly more frequent with active treatment (RR 1.14, 1.06–1.24), while serious adverse events and discontinuations did not clearly increase. The most robust evidence came from replicated phase 3 programs of dupilumab and nemolizumab.

**Conclusion:**

Replicated randomized evidence supports clinically meaningful improvements in itch and lesion outcomes with dupilumab and nemolizumab. Symptomatic benefits may emerge earlier than lesion responses, but this pattern should be interpreted cautiously because evidence across time points derives from limited, partly non-overlapping trial sets. Comparative treatment ranking is not supported, and longer-term data are needed to define rare, delayed, and agent-specific harms.

**Systematic Review Registration:**

https://www.crd.york.ac.uk/prospero/display_record.php?ID=CRD420261445753, identifier CRD420261445753.

## Introduction

Prurigo nodularis (PN) is a chronic neuroimmune inflammatory skin disease characterized by intense itch and multiple hyperkeratotic nodules (1). Persistent scratching sustains the itch–scratch cycle and contributes to sleep disturbance, impaired quality of life (2), and psychological burden (3). Current evidence places abnormal crosstalk among sensory neurons, immune cells, keratinocytes, and dermal stromal cells at the center of disease pathogenesis (4). This framework has shifted treatment development away from nonspecific immunosuppression toward pathway-directed systemic therapy (5).

A growing range of systemic targeted therapies has entered clinical development for PN (6). These agents act on pruritus-related immune, neural, and neuroimmune pathways involved in itch signaling, inflammation, and lesion persistence. Dupilumab blocks IL-4R*α* signaling, nemolizumab targets IL-31RA, and vixarelimab inhibits OSMR*β*. Other agents target KIT, neurokinin-1 receptors, or *κ*- and *μ*-opioid receptors (7). The evidence base differs substantially across treatments. Dupilumab and nemolizumab have been evaluated in repeated phase 3 programs, whereas vixarelimab, barzolvolimab, serlopitant, and extended-release nalbuphine remain supported mainly by early-phase or single-trial evidence (8). Their clinical effects may also emerge at different times and across different domains of disease burden.

Previous systematic reviews have summarized efficacy evidence for established therapies (9, 10), while a network meta-analysis has explored indirect comparisons among active treatments (11). The remaining clinical uncertainty is therefore not simply whether targeted therapy can be effective, but which conclusions are supported by replicated phase 3 evidence, which remain preliminary, and at what follow-up times different outcome domains have been demonstrated. Direct randomized evidence for emerging agents has not been synthesized consistently across follow-up times, response definitions, analysis populations, and levels of certainty. Without these distinctions, pooled estimates may obscure substantial differences in evidence maturity and clinical interpretability.

We therefore conducted a systematic review and pairwise meta-analysis of randomized controlled trials evaluating systemic targeted therapies for PN. Compatible placebo-controlled outcomes were synthesized separately at prespecified weeks 4, 8, 12, 16, and 24, without treating follow-up times or outcome definitions as interchangeable. Our objective was to distinguish replicated evidence from early therapeutic signals, clarify the follow-up times and clinical domains in which benefits have been demonstrated, and define the limits of current efficacy, comparative, and safety inferences.

## Methods

### Protocol and registration

This systematic review was conducted in accordance with the Cochrane Handbook for Systematic Reviews of Interventions (12) and reported according to the PRISMA 2020 statement (13). The review question and eligibility criteria were developed using the PICOTS framework. The protocol was registered in the International Prospective Register of Systematic Reviews (PROSPERO; registration number CRD420261445753).

### Search strategy

We searched PubMed, Embase.com, Web of Science Core Collection, the Cochrane Central Register of Controlled Trials (CENTRAL), and ClinicalTrials.gov from inception through June 30, 2026. Search strategies combined free-text terms and, where applicable, controlled vocabulary for prurigo nodularis or chronic nodular prurigo with the names, development codes, and molecular targets of systemic therapies directed at pruritus-related immune, neural, and neuroimmune pathways. Search terms covered dupilumab, nemolizumab, vixarelimab, barzolvolimab, serlopitant, extended-release nalbuphine, and relevant investigational targets, together with terms for randomized and placebo-controlled trials. We also screened the reference lists of included trials and relevant systematic reviews and performed forward citation tracking of eligible reports. No language restrictions were applied. Full database-specific search strategies are provided in Supplementary Table S1.

### Inclusion and exclusion criteria

We included parallel-group randomized controlled trials enrolling patients with an investigator-confirmed diagnosis of prurigo nodularis, nodular prurigo, or chronic nodular prurigo. Trials primarily enrolling patients with atopic dermatitis and describing only a prurigo-like phenotype were excluded, unless data for a separately diagnosed prurigo nodularis subgroup were independently extractable. Coexisting atopic dermatitis or an atopic background was permitted when prurigo nodularis was the target disease. The evidence base was predominantly adult. Trials allowing background topical therapy were eligible when the regimen was protocolized and applied consistently across randomized groups. Studies were required to report at least one extractable randomized comparative efficacy or safety outcome.

Independent randomized cohorts reported within the same article were treated as separate trials when their participants did not overlap. Multiple publications, supplementary reports, and registry records relating to the same randomized trial were linked and evaluated as a single study. We excluded nonrandomized studies, uncontrolled open-label extensions, studies with ineligible populations, interventions, or comparators, and reports without extractable randomized comparative data. Outcomes reported exclusively during open-label extension periods were not included in the randomized efficacy or safety meta-analyses.

### Outcome definitions

The primary efficacy outcome was the proportion of participants achieving a ≥ 4-point reduction from baseline in the weekly average Worst Itch Numerical Rating Scale (WI-NRS) (14) or Peak Pruritus Numerical Rating Scale (PP-NRS) (15) at weeks 4, 8, 12, 16, and 24. These time points were prespecified, and the two scales were considered clinically comparable because both assess peak or worst itch intensity.

Secondary outcomes included percentage and absolute changes from baseline in itch NRS, Investigator's Global Assessment (IGA) success, same-patient dual response in itch and lesions, a ≥ 4-point improvement in the Sleep Disturbance Numerical Rating Scale, and changes in the Dermatology Life Quality Index (DLQI), Hospital Anxiety and Depression Scale–Anxiety subscale (HADS-A), and Hospital Anxiety and Depression Scale–Depression subscale (HADS-D). IGA success was defined according to the original trial as an IGA or IGA for prurigo nodularis stage (IGA PN-S) score of 0 or 1, or as an IGA score of 0 or 1 accompanied by a ≥ 2-grade improvement from baseline. These trial-specific definitions were retained in the primary analysis. Dual response was defined as the simultaneous achievement, by the same participant at the same follow-up time point, of both a ≥ 4-point reduction in WI-NRS or PP-NRS and trial-defined IGA success. Dual-response data were included only when this composite outcome was reported directly for the same participants. Patient-reported outcomes were pooled only when the scales, outcome definitions, follow-up times, and analysis populations were sufficiently comparable; otherwise, they were summarized narratively.

Safety outcomes included treatment-emergent adverse events, serious adverse events, adverse events leading to treatment discontinuation, deaths, and prespecified adverse events of clinical interest. Open-label extension data were excluded. Full outcome definitions and harmonization rules are provided in Supplementary Table S2.

### Study selection and data extraction

Two reviewers (Y.Y. and S.L.) independently screened titles and abstracts, assessed potentially eligible full-text reports, and extracted data using a standardized form. Disagreements were resolved through discussion or, when necessary, consultation with a third reviewer (W.C.). Extracted information included publication and trial characteristics, country or region, study design and phase, registration details, eligibility criteria, randomized sample size, intervention target and dose, comparator and background therapy, follow-up duration, baseline participant characteristics, analysis population, outcome definitions, and efficacy and safety data. In multi-arm trials, data were extracted separately for each eligible dose. Treatment arms were combined for the primary treatment-versus-control analysis, with the shared control group counted once; dose-specific data were also retained. For dichotomous outcomes, the number of participants with the event and the total number analysed in each randomized group were extracted at each eligible follow-up time. For continuous outcomes, means and standard deviations were extracted preferentially. Adjusted between-group differences in change from baseline with 95% confidence intervals were used when reported. When means or standard deviations were unavailable, they were derived from standard errors, confidence intervals, medians, ranges, or interquartile ranges using Cochrane guidance and validated methods, provided that the required assumptions were reasonable (16, 17). All data conversions and their sources were documented.

### Quality assessment

Risk of bias was independently assessed by two reviewers (Y.Y. and S.L.) using the Cochrane RoB 2 tool (18), covering the randomization process, deviations from intended interventions, missing outcome data, outcome measurement, selective reporting, and overall bias. Judgments were classified as low risk, some concerns, or high risk, and were summarized using traffic-light and weighted summary plots. The certainty of evidence for each prespecified outcome and follow-up time point was evaluated using GRADE (19), considering risk of bias, inconsistency, indirectness, imprecision, and publication bias. Evidence certainty was rated as high, moderate, low, or very low. Disagreements were resolved through discussion or consultation with a third reviewer (W.C.). Imprecision judgments considered the width and clinical implications of the 95% CI, the total number of participants or events, and whether the optimal information size was met; the number of contributing studies alone was not used as a reason for downgrading.

### Statistical analysis

Dichotomous outcomes were summarized as risk ratios (RRs) and continuous outcomes measured on the same scale as mean differences (MDs), each with 95% confidence intervals (CIs) (12). Binary data were pooled using Mantel–Haenszel methods. Continuous outcomes and adjusted between-group estimates were synthesized using inverse-variance and generic inverse-variance methods, respectively. Random-effects models were used for the primary analyses because clinical heterogeneity was anticipated across therapies, trial phases, dose regimens, and study populations. Between-study variance was estimated using the DerSimonian–Laird method (20). Because most outcome-specific meta-analyses included only two to four trials, estimates of between-study heterogeneity and conventional random-effects confidence intervals were recognized as potentially unstable. We therefore interpreted pooled estimates with particular attention to the direction and magnitude of effect, confidence-interval width, consistency across contributing trials, and certainty of evidence, rather than relying solely on statistical significance or the observed I^2^ value. All analyses were performed using Review Manager version 5.4 (21).

Single-zero studies were included using RevMan's default handling of sparse data. Double-zero studies did not contribute to pooled RRs but were retained in descriptive summaries. Heterogeneity was assessed using I^2^ (22) and *τ*^2^ (23) and interpreted in relation to the number and size of contributing trials, the direction of effects, and clinical and methodological differences. Negative MDs favored active treatment for itch severity, DLQI, HADS-A, and HADS-D. RRs greater than 1 favored active treatment for efficacy outcomes and indicated more events for safety outcomes.

Sensitivity analyses examined stricter IGA definitions (an IGA score of 0 or 1 and a ≥ 2-grade improvement from baseline), restriction to replicated drug programmes, alternative dose selection in multi-arm trials, exclusion of clinically distinct or very small early-phase trials, fixed-effect modelling, and restriction to trials at low overall risk of bias. Exploratory subgroup analyses were conducted by drug or mechanism when clinically interpretable groups were available. Subgroup differences were evaluated using formal interaction tests (12). Statistical significance within one subgroup but not another was not considered evidence of effect modification. Single-trial subgroups and dose-specific analyses with shared controls were interpreted descriptively and not as head-to-head comparisons.

Funnel plots were examined for outcomes with at least 10 trials and were considered exploratory because of the limited number of studies and sparse events (24).

## Results

### Study selection and characteristics

The searches identified 721 records: 403 from PubMed, 192 from Embase, 67 from Web of Science, 34 from CENTRAL, and 25 from ClinicalTrials.gov. After removal of 236 duplicates, 485 records underwent title and abstract screening. Following full-text assessment, 10 reports representing 11 independent randomized controlled trials were included. The main reasons for full-text exclusion were non-randomized design, ineligible populations or interventions, absence of extractable randomized comparative data, duplicate or secondary reports, and duplicate or unpublished trial registry records. The study selection process is shown in Figure 1.

**Figure 1:**
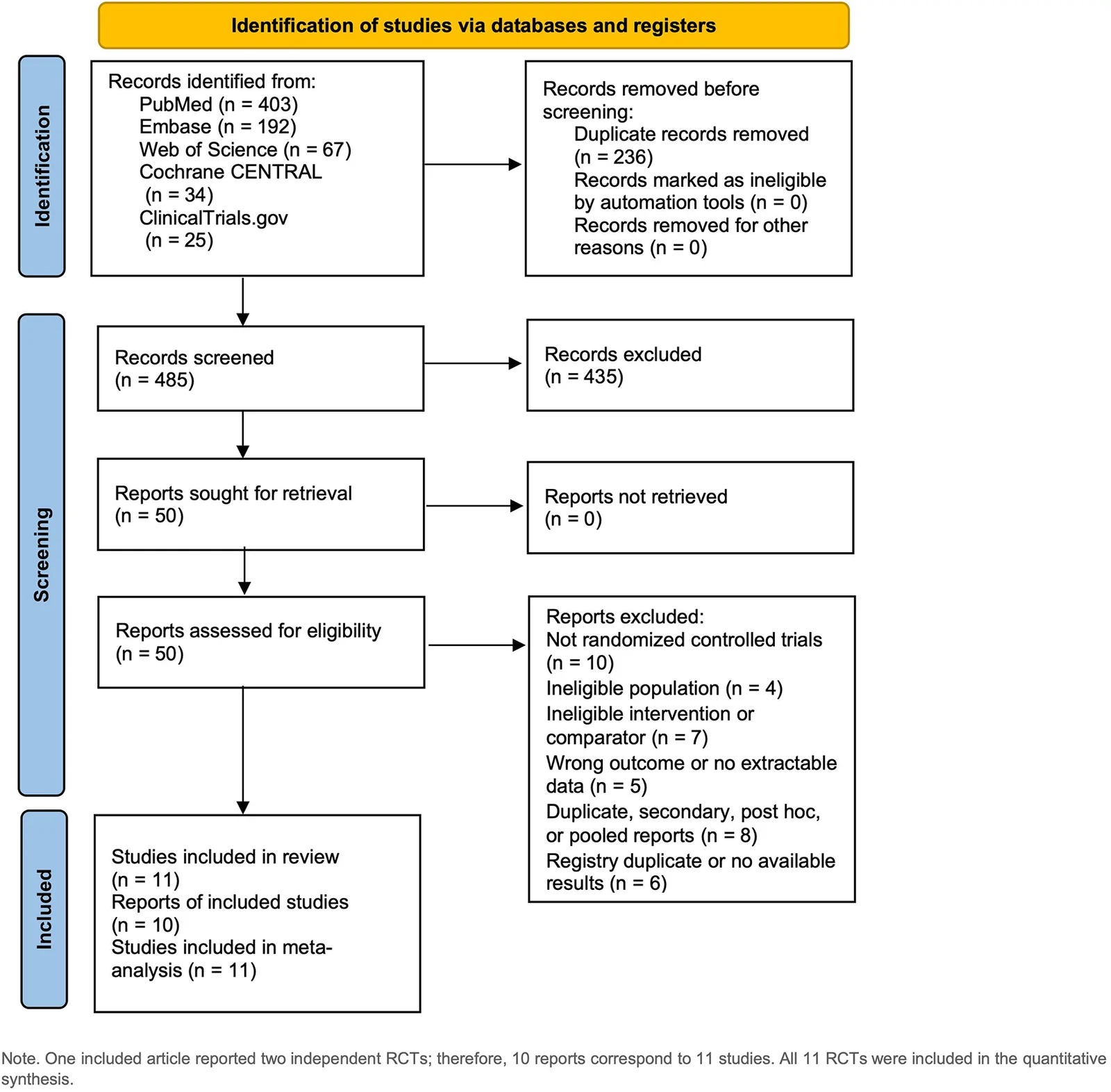
PRISMA flow diagram of study selection.

The 11 trials randomized 1,623 participants, of whom 1,622 received at least one dose of the assigned treatment. Four evaluated nemolizumab (25–28), two dupilumab (29), two vixarelimab (30, 31), and one each evaluated barzolvolimab (32), serlopitant (33), and extended-release nalbuphine (34). Sample sizes ranged from 23 to 286 participants, and randomized controlled periods ranged from approximately 8–24 weeks. Detailed design and intervention characteristics are presented in Table 1. Most trials enrolled predominantly adult patients with moderate-to-severe prurigo nodularis. One Japanese nemolizumab trial enrolled participants aged ≥13 years and used protocol-defined stable background topical therapy in all randomized groups. Group-specific baseline characteristics are provided in Table 2.

**Table 1:** Design and intervention characteristics of included randomized trials.

Study (trial)	Country/region	Trial registration	Randomized, n	Design	Key eligibility criteria	Active intervention(s)	Comparator	Treatment/follow-up	Therapeutic target
Ständer 2025 (phase 2b vixarelimab)	USA, Canada, Europe, and Asia	NCT03816891	190	Phase 2b; multicentre, randomized, double-blind, placebo-controlled, four-arm trial	Adults aged 18–80 years; PN ≥6 months; ≥20 pruritic nodules; WI-NRS ≥7; PN-IGA ≥3	Vixarelimab 120, 360, or 540 mg SC q4w	Placebo SC q4w	16 weeks	OSMR*β* monoclonal antibody
Metz 2025	Multinational	NCT04944862	23	Phase 1b; randomized, placebo-controlled, single-dose trial	Adults with moderate-to-severe PN	Barzolvolimab 1.5 or 3 mg/kg IV, single dose	Placebo IV, single dose	24 weeks	Anti-KIT monoclonal antibody
Yosipovitch 2023 (PRIME)	Multinational	NCT04183335	151	Phase 3; multicentre, randomized, double-blind, placebo-controlled, parallel-group trial	Adults with ≥20 nodules and severe itch inadequately controlled with topical therapy	Dupilumab 300 mg SC q2w plus stable TCS/TCI	Placebo SC q2w plus stable TCS/TCI	24 weeks	IL-4R*α* monoclonal antibody
Yosipovitch 2023 (PRIME2)	Multinational	NCT04202679	160	Phase 3; multicentre, randomized, double-blind, placebo-controlled, parallel-group trial	Adults with ≥20 nodules and severe itch inadequately controlled with topical therapy	Dupilumab 300 mg SC q2w plus stable TCS/TCI	Placebo SC q2w plus stable TCS/TCI	24 weeks	IL-4Rα monoclonal antibody
Ständer 2025 (OLYMPIA 1)	Multinational	NCT04501666	286	Phase 3; multicentre, randomized, double-blind, placebo-controlled trial	Adults with moderate-to-severe PN; PN ≥6 months; ≥20 nodules; IGA ≥3; PP-NRS ≥7	Nemolizumab SC q4w: 60-mg loading dose, then 30 mg (<90 kg) or 60 mg (≥90 kg)	Placebo SC q4w	24 weeks	IL-31RA monoclonal antibody
Sofen 2023	USA and Canada	NCT03816891	49	Phase 2a; randomized, double-blind, placebo-controlled trial	Adults with moderate-to-severe PN; PN ≥6 months; ≥10 nodules; WI-NRS ≥7	Vixarelimab 720-mg loading dose, then 360 mg SC weekly	Placebo SC weekly	8 weeks	OSMRβ monoclonal antibody
Ständer 2020	Multinational	NCT03181503	70	Phase 2; randomized, double-blind, placebo-controlled trial	Moderate-to-severe PN; ≥20 nodules; severe pruritus; PP-NRS ≥7	Nemolizumab 0.5 mg/kg SC at baseline and weeks 4 and 8	Placebo SC at baseline and weeks 4 and 8	12 weeks	IL-31RA monoclonal antibody
Ständer 2019	Germany	NCT02196324	128	Phase 2; randomized, double-blind, placebo-controlled trial	Treatment-refractory PN >6 weeks; pruritus VAS ≥7 cm	Serlopitant 5 mg orally once daily	Placebo orally once daily	8 weeks	Neurokinin-1 receptor antagonist
Kwatra 2023 (OLYMPIA 2)	Multinational	NCT04501679	274	Phase 3; multicentre, randomized, double-blind, placebo-controlled trial	Adults with moderate-to-severe PN; PN ≥6 months; ≥20 bilateral nodules; IGA 3/4; PP-NRS ≥7	Nemolizumab SC q4w: 60-mg loading dose, then 30 mg (<90 kg) or 60 mg (≥90 kg)	Placebo SC q4w	16-week treatment; 24-week follow-up	IL-31RA monoclonal antibody
Yokozeki 2024	Japan	jRCT2011200017	229	Phase 2/3; multicentre, randomized, double-blind, placebo-controlled, parallel-group trial	Age ≥13 years; PN ≥6 months; ≥20 bilateral nodules; PP-NRS ≥7; inadequate response to TCS and antihistamines	Nemolizumab 30 or 60 mg SC q4w plus stable TCS/TCI	Placebo SC q4w plus stable TCS/TCI	16 weeks	IL-31RA monoclonal antibody
Weisshaar 2022	North America and Europe	NCT02174419	63	Phase 2; multicentre, randomized, double-blind, placebo-controlled trial	Adults with PN; chronic pruritus ≥6 weeks; lesions in ≥2 anatomical areas; WI-NRS ≥5	Nalbuphine ER 81 or 162 mg orally twice daily	Placebo orally twice daily	10 weeks	*κ*-opioid receptor agonist and μ-opioid receptor antagonist
									

Sofen 2023 and Ständer 2025 shared NCT03816891 but represented distinct phase 2a and phase 2b cohorts with different dosing regimens, treatment durations, and non-overlapping enrolment periods; they were therefore treated as separate study cohorts.

BID, twice daily; IGA, investigator's global assessment; IL-4Rα, interleukin-4 receptor α; IL-31RA, interleukin-31 receptor A; IV, intravenous; OSMRβ, oncostatin M receptor β; PN, prurigo nodularis; PO, orally; PP-NRS, peak pruritus numerical rating scale; q2w, every 2 weeks; q4w, every 4 weeks; SC, subcutaneous; TCI, topical calcineurin inhibitor; TCS, topical corticosteroid; VAS, visual analogue scale; WI-NRS, worst itch numerical rating scale.

**Table 2:** Baseline demographic and clinical characteristics by randomized group.

Study (trial)	Randomized group	n	Age, years	Female, *n* (%)	Disease duration	Baseline itch intensity	IGA grade 3/4, n	Baseline sleep score	DLQI	HADS-A/HADS-D
Ständer 2025 (phase 2b vixarelimab)	Vixarelimab 540 mg	47	56.7 ± 12.2	29 (61.7%)	≥10 y: 20	WI-NRS 8.4 ± 0.9	NR	NR	NR	NR
	Vixarelimab 360 mg	47	55.9 ± 13.7	28 (59.6%)	≥10 y: 19	WI-NRS 8.7 ± 0.9	NR	NR	NR	NR
	Vixarelimab 120 mg	47	54.4 ± 16.1	28 (59.6%)	≥10 y: 15	WI-NRS 8.4 ± 1.2	NR	NR	NR	NR
	Placebo	48	54.8 ± 13.4	29 (60.4%)	≥10 y: 20	WI-NRS 8.6 ± 0.9	NR	NR	NR	NR
Metz 2025	Barzolvolimab 1.5 mg/kg	7	63.7, median	4 (57.1%)	8.1 y, median	WI-NRS 8.6	NR	NR	NR	NR
	Barzolvolimab 3 mg/kg	8	54.8, median	6 (75.0%)	6.0 y, median	WI-NRS 8.5	NR	NR	NR	NR
	Placebo	8	51.9, median	2 (25.0%)	9.6 y, median	WI-NRS 8.7	NR	NR	NR	NR
Yosipovitch 2023 (PRIME)	Dupilumab	75	49.2 ± 17.4	52 (69.3%)	6.0 ± 7.6 y	WI-NRS 8.6 ± 0.9	54/21	Sleep NRS 4.4 ± 2.4	17.8 ± 7.1	8.5 ± 5.2/6.0 ± 3.8
	Placebo	76	51.1 ± 15.8	48 (63.2%)	5.4 ± 6.2 y	WI-NRS 8.3 ± 1.1	53/22	Sleep NRS 4.3 ± 2.2	15.7 ± 7.3	8.3 ± 4.6/6.0 ± 4.1
Yosipovitch 2023 (PRIME2)	Dupilumab	78	51.0 ± 15.8	52 (66.7%)	5.4 ± 6.9 y	WI-NRS 8.5 ± 1.0	49/29	Sleep NRS 4.4 ± 2.3	18.2 ± 6.5	9.3 ± 4.2/6.9 ± 4.0
	Placebo	82	46.7 ± 15.2	51 (62.2%)	5.5 ± 7.0 y	WI-NRS 8.5 ± 1.0	49/32	Sleep NRS 4.2 ± 2.5	18.2 ± 7.0	9.5 ± 5.1/6.3 ± 4.0
Ständer 2025 (OLYMPIA 1)	Nemolizumab	190	57.5 ± 12.8	110 (57.9%)	86.9 ± 85.3 mo	PP-NRS 8.5 ± 0.9	107/83	SD-NRS 7.0 ± 2.4	17.1 ± 7.0	7.8 ± 4.5/6.7 ± 4.7
	Placebo	96	57.6 ± 13.4	56 (58.3%)	100.6 ± 98.6 mo	PP-NRS 8.4 ± 1.0	62/34	SD-NRS 6.9 ± 2.3	16.9 ± 6.7	7.3 ± 4.4/6.5 ± 4.7
Sofen 2023	Vixarelimab	23	52.0 ± 15.56	13 (56.5%)	12.2 ± 13.32 y	WI-NRS 8.7 ± 0.88	13/9	Sleep NRS 7.6 ± 2.2	16.1 ± 7.99	NR
	Placebo	26	64.1 ± 7.86	16 (61.5%)	17.2 ± 14.89 y	WI-NRS 8.0 ± 1.09	14/12	Sleep NRS 7.0 ± 1.5	14.8 ± 7.07	NR
Ständer 2020	Nemolizumab	34	59.7 ± 13.2	19 (55.9%)	NR	PP-NRS 8.4	16/18	SD-NRS 7.4, median	NR	NR
	Placebo	36	52.4 ± 17.5	22 (61.1%)	NR	PP-NRS 8.4	22/14	SD-NRS 6.8, median	NR	NR
Ständer 2019	Serlopitant	65	57.10 ± 12.00	31 (47.7%)	>10 y: 18	Average itch NRS 7.60 ± 1.46	NR	NR	NR	NR
	Placebo	63	58.1 ± 11.14	36 (57.1%)	>10 y: 19	Average itch NRS 7.65 ± 1.67	NR	NR	NR	NR
Kwatra 2023 (OLYMPIA 2)	Nemolizumab	183	53.7 ± 14.4	113 (61.7%)	104 ± 100.7 mo	PP-NRS 8.5 ± 0.6	108/75	SD-NRS 7.2 ± 2.2	16.5 ± 6.8	8.1 ± 4.5/6.6 ± 4.2
	Placebo	91	50.8 ± 15.0	56 (60.4%)	108 ± 114.9 mo	PP-NRS 8.5 ± 1.0	48/43	SD-NRS 7.3 ± 2.2	17.1 ± 6.6	7.2 ± 4.2/5.4 ± 4.0
Yokozeki 2024	Nemolizumab 30 mg	77	50, median	40 (51.9%)	3.3 y, median	PP-NRS 8.6, median(6.4–10.0)	35/42	SD-NRS 7.9, median	12, median	8.14 ± 2.93/7.82 ± 4.2
	Nemolizumab 60 mg	76	51, median	42 (55.3%)	4.3 y, median	PP-NRS 8.4, median(6.7–10.0)	36/40	SD-NRS 7.6, median	12, median	7.17 ± 3.55/5.89 ± 4.11
	Placebo	76	54, median	40 (52.6%)	4.8 y, median	PP-NRS 8.4, median(7.0–10.0)	41/35	SD-NRS 8.0, median	11, median	7.36 ± 4.91/5.55 ± 3.35
Weisshaar 2022	Nalbuphine ER 81 mg BID	22	55.0 ± 8.6	12 (54.5%)	>10 y: 8	Average itch NRS 7.72 ± 1.38	NR	NR	NR	NR
	Nalbuphine ER 162 mg BID	19	49.1 ± 16.3	10 (52.6%)	>10 y: 7	Average itch NRS 7.28 ± 1.54	NR	NR	NR	NR
	Placebo	22	53.3 ± 16.7	12 (54.5%)	>10 y: 5	Average itch NRS 7.26 ± 1.39	NR	NR	NR	NR

IGA values are reported as the number of participants with grade 3/grade 4 disease where available.

DLQI, dermatology life quality index; HADS-A/D, hospital anxiety and depression scale—anxiety/depression; IGA, investigator's global assessment; NR, not reported; PP-NRS, peak pruritus numerical rating scale; SD-NRS, sleep disturbance numerical rating scale; WI-NRS, worst itch numerical rating scale.

### Risk of bias

All 11 trials were assessed using RoB 2. No trial was judged to be at high overall risk of bias. The remaining trials were rated as having low risk or some concerns, mainly because of missing outcome data and selection of the reported result. Detailed assessments are shown in Supplementary Figures S1, S2.

### Efficacy outcomes

Forest plots for the primary itch-response outcome are presented in Figure 2, and those for IGA success and same-patient dual response in Figure 3. Supplementary Table S3 provides the other efficacy results for continuous itch, sleep, quality-of-life, and psychological outcomes. Supplementary Table S4 offers an overview of the availability and direction of findings across prespecified follow-up times, whereas Supplementary Table S5 presents the corresponding pooled estimates for the safety.

**Figure 2:**
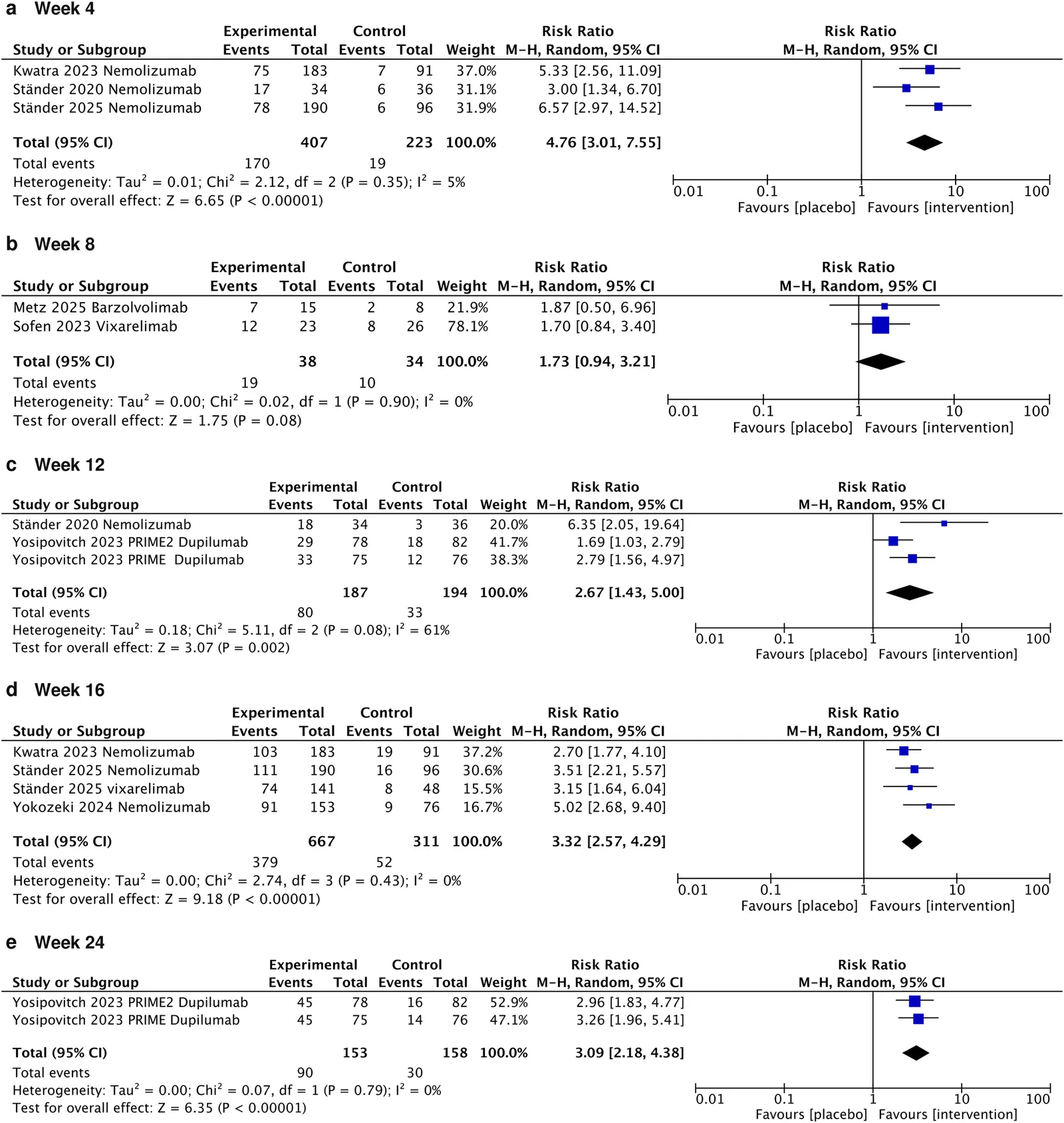
Forest plots of WI-NRS/PP-NRS ≥4-point itch response across follow-up times: **(a)** week 4, **(b)** week 8, **(c)** week 12, **(d)** week 16, and **(e)** week 24.

**Figure 3:**
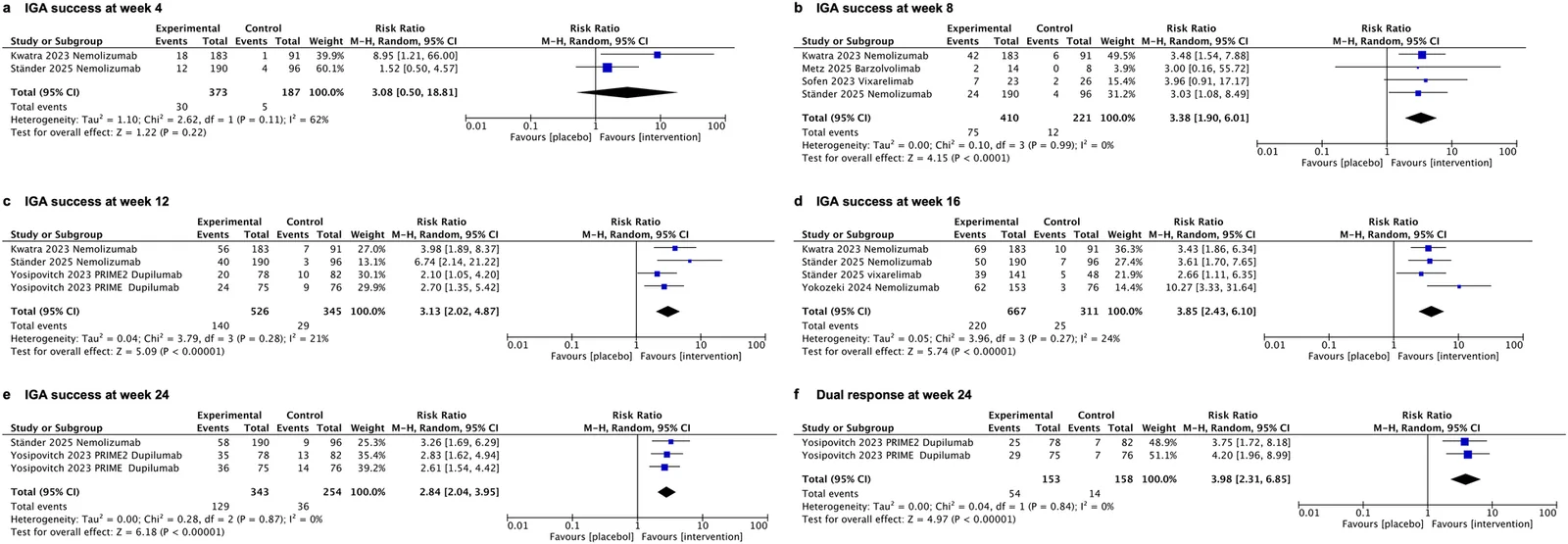
Forest plots of IGA success and dual response across follow-up times. Pooled risk ratios are shown for IGA success at **(a)** week 4, **(b)** week 8, **(c)** week 12, **(d)** week 16, and **(e)** week 24, and for dual response at **(f)** week 24.

### Itch outcomes

Across the trials contributing to each follow-up time, active treatment was associated with a higher proportion of participants achieving a ≥ 4-point reduction in WI-NRS or PP-NRS at week 4 (3 trials, 630 participants; RR 4.76, 95% CI 3.01–7.55; I^2^ = 5%). At week 8, the analysis included two early-phase trials with 72 participants. The point estimate favored treatment, but the confidence interval crossed the null (RR 1.73, 95% CI 0.94–3.21; I^2^ = 0%). Higher response rates were observed at week 12 (3 trials, 381 participants; RR 2.67, 95% CI 1.43–5.00; I^2^ = 61%), week 16 (4 trials, 978 participants; RR 3.32, 95% CI 2.57–4.29; I^2^ = 0%), and week 24 (2 trials, 311 participants; RR 3.09, 95% CI 2.18–4.38; I^2^ = 0%).

Continuous itch outcomes were generally consistent with the primary response analysis. Percentage change from baseline in weekly average peak or worst itch NRS favored treatment at week 4 (MD −31.91 percentage points, 95% CI −38.49 to −25.33; I^2^ = 0%), week 8 (MD −30.79, 95% CI −41.66 to −19.92; I^2^ = 28%), week 12 (MD −34.44, 95% CI −41.86 to −27.03; I^2^ = 0%), and week 24 (MD −30.21, 95% CI −40.58 to −19.83; I^2^ = 67%). Absolute change in itch NRS also favored treatment at week 8 (MD −2.12 points, 95% CI −3.34 to −0.90; I^2^ = 56%) and week 12 (MD −2.92, 95% CI −3.54 to −2.30; I^2^ = 0%).

### Lesion response and dual response

At week 4, the estimate for IGA success was imprecise and crossed the null (2 trials, 560 participants; RR 3.08, 95% CI 0.50–18.81; I^2^ = 62%). Separate analyses at later follow-up times showed higher IGA success rates with treatment at week 8 (4 trials, 631 participants; RR 3.38, 95% CI 1.90–6.01; I^2^ = 0%), week 12 (4 trials, 871 participants; RR 3.13, 95% CI 2.02–4.87; I^2^ = 21%), week 16 (4 trials, 978 participants; RR 3.85, 95% CI 2.43–6.10; I^2^ = 24%), and week 24 (3 trials, 597 participants; RR 2.84, 95% CI 2.04–3.95; I^2^ = 0%).

Two phase 3 dupilumab trials reported same-patient dual response at week 24. A combined itch and lesion response was achieved by 54 of 153 participants receiving dupilumab and 14 of 158 receiving placebo, corresponding to an RR of 3.98 (95% CI 2.31–6.85; I^2^ = 0%). These outcomes are shown in Figure 3.

### Sleep, quality of life, and psychological outcomes

Sleep response data were available from nemolizumab trials. A ≥ 4-point improvement in SD-NRS was more frequent with treatment at week 4 (2 trials, 560 participants; RR 4.42, 95% CI 2.62–7.45; I^2^ = 0%) and week 16 (3 trials, 754 participants; RR 3.04, 95% CI 2.23–4.15; I^2^ = 18%). Other studies used sleep-loss visual analogue scales, difficulty-falling-asleep NRS, or MOS-R measures and were not pooled with SD-NRS response because of differences in instruments and outcome definitions.

DLQI favored treatment at week 16 (2 nemolizumab trials, 560 participants; MD −7.27 points, 95% CI −8.99 to −5.56; I^2^ = 32%) and week 24 (3 trials, 597 participants; MD −6.96, 95% CI −8.37 to −5.56; I^2^ = 27%). At week 24, HADS-A and HADS-D analyses included the same three trials and 597 participants. The pooled MD was −1.51 points for HADS-A (95% CI −2.11 to −0.92; I^2^ = 0%) and −1.55 points for HADS-D (95% CI −2.08 to −1.02; I^2^ = 0%).

### Safety outcomes

All 11 trials, comprising 1,622 participants, reported treatment-emergent adverse events. Across the included randomized periods, treatment-emergent adverse events were more frequent with active treatment than with placebo (RR 1.14, 95% CI 1.06–1.24; I^2^ = 0%). No statistically significant difference was observed for serious adverse events (RR 0.72, 95% CI 0.48–1.10; I^2^ = 0%) or discontinuation due to adverse events (10 trials, 1,565 participants; RR 1.08, 95% CI 0.61–1.91; I^2^ = 0%).

Confidence intervals crossed the null for all prespecified specific adverse events, including conjunctivitis (3 trials, 379 participants; RR 1.71, 95% CI 0.53–5.57; I^2^ = 0%), headache (8 trials, 1,173 participants; RR 1.37, 95% CI 0.83–2.25; I^2^ = 0%), edema (3 trials, 683 participants; RR 2.33, 95% CI 0.73–7.45; I^2^ = 0%), and eczematous adverse events (5 trials, 961 participants; RR 2.09, 95% CI 0.76–5.71; I^2^ = 15%). One death occurred in a placebo group and was considered unrelated to study treatment; no other deaths were reported.

### Evidence by intervention

Intervention-level findings are summarized in Table 3. Dupilumab and nemolizumab were supported by replicated phase 3 programmes and contributed most of the evidence for clinically meaningful itch and IGA responses. Nemolizumab also provided the pooled evidence for SD-NRS response; same-patient dual response was reported only in the two phase 3 dupilumab trials. Vixarelimab showed favorable findings across itch, lesion, sleep, and quality-of-life measures, although the evidence was limited to two trials and remained sensitive to dose selection in the multi-arm study. Barzolvolimab was evaluated in a very small early-phase trial and provided primarily proof-of-concept evidence. Serlopitant showed favorable between-group differences in mean itch VAS at weeks 4 and 8. Extended-release nalbuphine did not show a clear benefit in the primary modified intention-to-treat analysis, while favorable itch and ItchyQoL findings were confined to a high-dose completer analysis. Drug-specific safety data for the emerging therapies were sparse and based on short randomized periods, preventing reliable assessment of uncommon or delayed adverse events.

**Table 3:** Summary of efficacy and safety evidence by intervention.

Intervention	Pruritus	Skin lesion response	Sleep	Dual response	Health-related quality of life	Psychological symptoms	Safety evidence	Overall evidence maturity
Dupilumab	Consistent benefit in ≥4-point NRS response at weeks 12 and 24; percentage change from baseline also favoured dupilumab at week 24.	Consistent improvement in IGA success at weeks 12 and 24.	A favourable change in sleep-related symptoms was reported at week 24, but no compatible SD-NRS ≥4-point responder estimate was available for quantitative pooling.	Demonstrated at week 24 in two phase 3 trials, supporting concurrent pruritus and lesion control within individual participants.	Consistent improvement in DLQI at week 24.	Small favourable changes in HADS-A and HADS-D at week 24; findings should be considered exploratory.	Relatively extensive safety reporting across two phase 3 trials; rare and treatment-specific adverse events remained imprecisely estimated.	Mature evidence base: replicated phase 3 trials with consistent multidomain efficacy at 12–24 weeks.
Nemolizumab	Consistent benefit at the principal reported follow-up times, particularly weeks 4, 12, 16, and 24.	Consistent improvement in IGA success at weeks 8, 12, 16, and 24; the week-4 estimate remained imprecise.	Improvement in SD-NRS ≥4-point response at weeks 4 and 16.	Not reported in the currently supplied dual-response analysis.	Consistent improvement in DLQI at weeks 16 and 24.	Small favourable changes in HADS-A and HADS-D at week 24; findings should be considered exploratory.	The largest safety dataset in this review among the included interventions; estimates for uncommon and treatment-specific adverse events remained imprecise.	Mature evidence base: multiple randomized trials with the broadest time-resolved and multidomain efficacy evidence.
Vixarelimab	Emerging evidence of benefit: continuous pruritus outcomes improved at week 8; the ≥4-point responder estimate was imprecise at week 8 but favoured vixarelimab at week 16.	Preliminary evidence at week 8 and a clearer IGA response at week 16.	Preliminary but statistically significant improvement in sleep-loss VAS and difficulty-falling-asleep NRS was observed in the phase 2a trial. Not pooled with SD-NRS because the instruments and responder definitions differed; compatible phase 2b comparative results were unavailable.	Not reported.	Preliminary but statistically significant ItchyQoL improvement was observed through week 8 in the phase 2a trial, with generally favourable DLQI findings. Not pooled because compatible numerical estimates were unavailable and the instruments differed; comparative phase 2b results were not reported.	Not reported.	Limited to two short-term trials; the duration of exposure and precision of safety estimates were insufficient for firm conclusions.	Emerging evidence base: replicated early-phase signals, but no confirmatory phase 3 evidence or comprehensive patient-reported outcome data.
Barzolvolimab	Preliminary week-8 signal; responder and absolute-change estimates favoured treatment but were highly imprecise.	Preliminary week-8 IGA signal with a very wide confidence interval.	Sleep was assessed using the 12-item MOS-R, but no compatible comparative estimate was available for pooling with SD-NRS outcomes.	Not reported.	Not reported.	Not reported.	Based on a single very small early-phase trial; safety estimates were extremely imprecise.	Preliminary evidence base: proof-of-concept findings only, with insufficient precision for firm efficacy or safety conclusions.
Nalbuphine ER	The primary modified intention-to-treat analysis was not statistically significant, although a favourable dose-dependent antipruritic signal was observed among week-10 completers receiving NAL-ER 162 mg. Results were not pooled because the responder thresholds, follow-up timing, and analysis population differed from the harmonized ≥4-point NRS analyses.	PAS was assessed, but no compatible randomized lesion-response estimate was available for pooling with IGA success outcomes.	Not reported.	Not reported.	An improvement in ItchyQoL was observed among week-10 completers receiving NAL-ER 162 mg, but this exploratory completer analysis was not pooled with DLQI outcomes.	HADS was assessed, but no compatible comparative estimate was available for pooling with the week-24 HADS analyses.	Limited to one short-term trial, with sparse events and imprecise estimates.	Uncertain evidence base: a small phase 2 trial showed a possible antipruritic and quality-of-life signal, primarily in high-dose completers, whereas the primary modified intention-to-treat analysis was not statistically significant.
Serlopitant	Significant reductions in average itch VAS were observed versus placebo at weeks 4 and 8. These results were not pooled because the outcome scale and reporting format differed from the harmonized WI-NRS/PP-NRS analyses.	Not reported.	Not reported.	Not reported.	Not reported.	Not reported.	Safety evidence was derived from a single trial and was insufficiently precise for treatment-specific conclusions.	Limited but positive evidence base: a single phase 2 RCT demonstrated a statistically significant antipruritic effect, but confirmatory trials and compatible multidomain outcome data were unavailable.

Pruritus: NRS responder, percentage change from baseline, and absolute change from baseline are complementary measures of the same clinical domain and were not treated as independent domains.

Evidence maturity: This qualitative classification reflects trial phase, replication, consistency across outcome domains, duration of follow-up, and precision. It is not a formal GRADE rating, which should be assigned separately for each intervention, outcome, and time point.

Not reported: Indicates that no relevant outcome result was available.

DLQI, dermatology life quality index; HADS-A, hospital anxiety and depression scale–anxiety; HADS-D, hospital anxiety and depression scale–depression; IGA, investigator's global assessment; NRS, numerical rating scale; SD-NRS, sleep disturbance numerical rating scale.

### Sensitivity analyses, subgroup analyses, and certainty of evidence

Sensitivity analyses are presented in Supplementary Table S6. Restriction to trials using the strict IGA definition produced similar results at week 8 (RR 3.30, 95% CI 1.74–6.26; I^2^ = 0%) and week 16 (RR 4.36, 95% CI 2.45–7.75; I^2^ = 37%). When the week 16 itch-response analysis was restricted to nemolizumab trials, the pooled RR was 3.41 (95% CI 2.46–4.73; I^2^ = 27%). Exclusion of the clinically distinct Japanese trial (28) yielded a week 16 sleep-response RR of 3.18 (95% CI 1.83–5.55; I^2^ = 54%). Restriction of the week 24 DLQI analysis to the two dupilumab trials produced an MD of −6.26 points (95% CI −7.72 to −4.80; I^2^ = 0%). Restriction of week 24 percentage itch change to the dupilumab programme yielded an MD of −24.78 percentage points (95% CI −32.67 to −16.89; I^2^ = 0%). After exclusion of the very small barzolvolimab trial, the week 8 absolute itch change was −2.58 points (95% CI −3.56 to −1.61; I^2^ = 36%). In exploratory subgroup analyses of week 12 IGA success, the RR was 4.65 for nemolizumab (95% CI 2.49–8.68; I^2^ = 0%) and 2.38 for dupilumab (95% CI 1.46–3.89; I^2^ = 0%). The test for subgroup differences was not statistically significant (*P* for interaction=0.099). Interaction *P* values for drug subgroups at weeks 16 and 24 were 0.377 and 0.636, respectively. In dose-selection analyses of the multi-arm vixarelimab trial (30) at week 16, the pooled RR for the primary itch response was 3.12 when the 120-mg arm was included (95% CI 2.19–4.44; I^2^ = 41%), 3.40 with the 360-mg arm (95% CI 2.63–4.40; I^2^ = 0%), and 3.44 with the 540-mg arm (95% CI 2.66–4.44; I^2^ = 0%). Drug-stratified analyses of secondary efficacy outcomes, dose-selection analyses of the week 16 itch response, and therapeutic-class subgroup analyses of safety outcomes are presented in Supplementary Tables S7–S9, respectively. The certainty of evidence for the primary itch-response outcome was high at weeks 4 and 16, moderate at weeks 8 and 24, and low at week 12. After reassessment of imprecision, percentage-change outcomes for itch severity at weeks 4, 8, and 12 and the absolute-change outcome at week 12 were rated as moderate-certainty evidence; the absolute-change outcome at week 8 was rated as low-certainty evidence because both inconsistency and imprecision were serious. The high-certainty rating for DLQI change at week 16 was retained because 560 participants contributed, the confidence interval was narrow and excluded no effect, and no serious concerns were identified in the other GRADE domains. Certainty for the remaining outcomes ranged from high to very low and is detailed in Supplementary Table S10 (19).

Funnel plots are shown in Supplementary Figures S3–S5. Visual inspection did not reveal a consistent pattern of asymmetry; however, the funnel plots were largely uninformative because of the small number of estimable studies and sparse events.

## Discussion

This systematic review synthesized direct placebo-controlled evidence from 11 randomized trials of six systemic targeted therapies for PN. The most robust efficacy evidence came from replicated phase 3 programmes of dupilumab and nemolizumab, which supported clinically meaningful improvements in itch and lesion outcomes, with additional benefits reported for sleep, dermatology-related quality of life, and selected psychological outcomes. Vixarelimab showed preliminary signals across several clinical domains, whereas evidence for barzolvolimab, serlopitant, and extended-release nalbuphine remained sparse or dependent on selected outcomes and analysis populations. Across separate trial sets, itch reductions were observed as early as week 4, while IGA success was supported from week 8 onward. Because different therapies and trials informed each follow-up time, these findings should not be interpreted as a common treatment trajectory or a class effect shared by all systemic targeted therapies.

Patient-reported outcomes suggest that treatment benefits may extend beyond itch intensity and visible lesion improvement. In the nemolizumab programmes, sleep response was evident by week 4 and remained favorable at week 16, whereas pooled improvements in DLQI were demonstrated at weeks 16 and 24 (2, 25–29). Earlier sleep benefits may reflect relatively rapid relief of nocturnal itch and interruption of the itch–scratch–sleep-disturbance cycle. By contrast, DLQI captures broader effects on daily activities, social participation, emotional well-being, and treatment burden; improvement in this measure may therefore reflect the cumulative functional consequences of sustained control of itch and skin lesions. However, the sleep and DLQI findings were informed by partly different trials, therapies, and follow-up times, and aggregate data cannot establish that early sleep improvement mediated later quality-of-life gains. Evidence for anxiety and depressive symptoms was substantially more limited and should be regarded as exploratory rather than evidence of a direct psychotropic effect. These findings support multidomain assessment of treatment response while highlighting the limited evidence available for psychological outcomes.

The temporal findings may also have clinical relevance, although they require cautious interpretation. In the better-developed trial programmes, symptomatic improvement appeared to be detectable before consistent lesion response. Early symptomatic change may therefore provide complementary information alongside later lesion-based assessments. However, this pattern was derived from limited and partly non-overlapping trial sets and was driven largely by the dupilumab and nemolizumab programmes; a consistent sequence of multidomain response across therapies cannot be established. Same-patient dual response may offer a clinically informative endpoint by capturing simultaneous improvement in itch and lesions, but it has so far been reported in relatively few trials.

Previous evidence syntheses have either evaluated conventional and biologic therapies broadly, focused on individual established agents, summarized randomized treatment outcomes, or used network meta-analysis to explore indirect comparisons (8–11). The present review differs by restricting synthesis to direct randomized placebo-controlled evidence for six systemic targeted therapies and by preserving prespecified follow-up times, outcome definitions, and analysis populations. It also distinguishes replicated phase 3 evidence from signals arising from single or early-phase trials and applies GRADE separately by outcome and time point. Accordingly, our aim was not to rank active treatments but to identify where findings are reproducible, where they remain preliminary, and where conclusions are sensitive to analytic choices. This distinction is clinically important because pooled estimates do not carry equal certainty across drugs, outcomes, or follow-up times, and the current evidence does not support comparative treatment selection.

The certainty and stability of the pooled estimates varied substantially. For the primary itch-response outcome, GRADE certainty ranged from low to high across follow-up times. The week 8 binary responder estimate was imprecise despite favorable continuous itch outcomes, potentially reflecting limited statistical power and information loss associated with dichotomization. Moderate-to-substantial statistical heterogeneity was observed in several analyses, including the week 12 itch reduction and selected continuous itch outcomes. Potential contributors include differences in treatment mechanisms, trial populations, background therapy, outcome implementation, and analytic methods; however, because most pooled analyses included only two to four trials, the sources of heterogeneity could not be reliably determined. Conversely, low I^2^ values should not be interpreted as evidence of clinical homogeneity across mechanistically distinct therapies, particularly given the limited power to detect between-study heterogeneity in sparse meta-analyses. Sensitivity analyses generally preserved the direction of the main efficacy findings, and no clear subgroup interactions were detected; drug- and dose-specific subgroup findings should therefore remain exploratory rather than be interpreted as evidence of comparative efficacy or dose–response effects. These revised ratings also emphasize that apparently precise effects from small bodies of evidence require explicit consideration of the optimal information size and should not be judged by the number of contributing studies alone.

The available safety evidence was more informative for short-term tolerability than for uncommon or treatment-specific harms. Treatment-emergent adverse events were modestly more frequent with active treatment, whereas no clear increase in serious adverse events or treatment discontinuations was observed. Estimates for individual adverse events were imprecise, and the apparent increase in eczematous events was not robust to risk-of-bias restriction. Differences in adverse-event patterns across treatment programmes may reflect their distinct targets and pharmacologic effects, but may also arise from route and dose, exposure duration, baseline atopic comorbidity, concomitant topical therapy, and trial-specific ascertainment or reporting. For example, conjunctivitis was concentrated in the IL-4R*α*-blockade programme, whereas headache and eczematous events were reported more often in some IL-31RA trials; these observations are biologically plausible in the context of pathway-specific effects but remain statistically uncertain (7, 25–31). Class-stratified analyses did not demonstrate significant subgroup interactions, and the absence of head-to-head trials precludes causal comparison of safety profiles. Current randomized evidence therefore does not indicate a clear excess of serious short-term harm but remains insufficient to characterize rare, delayed, cumulative, or treatment-specific risks.

Several limitations should be acknowledged. First, most outcome- and time-specific analyses included only two to four trials, limiting precision and the reliable investigation of heterogeneity. Different follow-up times were also informed by partly different treatment programmes, and some pooled estimates were dominated by a single programme, restricting direct interpretation of changes over time. Second, differences in IGA definitions, background topical therapy, missing-data methods, and analysis populations reduced cross-trial comparability. Sleep, DLQI, and HADS outcomes were inconsistently reported, limiting conclusions regarding broader patient-reported benefits. Third, aggregate data precluded investigation of individual-level treatment modifiers and within-patient relationships among itch, sleep, lesion response, and quality of life. One trial included adolescents without separately reported adult data, making the evidence predominantly, but not exclusively, applicable to adults. Finally, the absence of active-comparator trials precluded conclusions regarding relative treatment superiority, and randomized follow-up of approximately 8–24 weeks was insufficient to assess sustained efficacy or uncommon and delayed adverse effects. Funnel-plot analyses were also underpowered and should be interpreted cautiously.

Despite these limitations, the review preserved prespecified follow-up times and avoided pooling incompatible outcome definitions, scales, or analysis populations. Same-patient dual response was included only when directly reported, and evidence certainty was assessed separately by outcome and follow-up time using GRADE. Sensitivity analyses addressed key clinical and methodological assumptions. These design features improve the clinical interpretability of the synthesis while providing a transparent distinction between replicated findings, preliminary signals, and areas in which uncertainty remains.

## Conclusion

Replicated phase 3 evidence provides the strongest support for clinically meaningful improvements in itch and lesion outcomes with dupilumab and nemolizumab, with additional benefits reported for selected patient-reported outcomes. Across the better-developed trial programmes, symptomatic improvement appeared to be detectable earlier than consistent lesion response, although the limited and partly non-overlapping evidence across follow-up times does not establish a uniform temporal response pattern across therapies. Vixarelimab has shown promising but preliminary findings, whereas evidence for barzolvolimab, serlopitant, and extended-release nalbuphine remains insufficient for firm conclusions. Current randomized evidence primarily informs short-term tolerability and does not permit reliable assessment of rare, delayed, or long-term harms. Future trials should harmonize outcome definitions and assessment times, report same-patient multidomain responses, incorporate active comparators, and extend safety follow-up.

## Data Availability

The original contributions presented in the study are included in the article/Supplementary Material, further inquiries can be directed to the corresponding author.
