# Supplementary material for: Direct randomized evidence for systemic targeted therapies in prurigo nodularis: a systematic review and meta-analysis
Source: Front Med (Lausanne). 2026 Oct 15;13:1942040. doi: 10.3389/fmed.2026.1942040 (PMC13717402; doi:10.3389/fmed.2026.1942040)
Supplement: Supplementary file 1 [file Table1.docx]

**Supplementary Table S1. Search strategies by database**

| **Database** | **Search strategy** |
| --- | --- |
| PubMed | ("prurigo nodularis"[tiab] OR "nodular prurigo"[tiab] OR "chronic nodular prurigo"[tiab] OR "chronic prurigo"[tiab] OR "Hyde's prurigo"[tiab] OR "prurigo of Hyde"[tiab] OR (prurigo[tiab] AND nodular*[tiab]) OR (Prurigo[Mesh] AND nodular*[tiab])) AND (dupilumab[tiab] OR nemolizumab[tiab] OR vixarelimab[tiab] OR barzolvolimab[tiab] OR serlopitant[tiab] OR nalbuphine[tiab] OR "nalbuphine extended-release"[tiab] OR "nalbuphine ER"[tiab] OR Haduvio[tiab] OR aprepitant[tiab] OR tradipitant[tiab] OR benralizumab[tiab] OR rocatinlimab[tiab] OR povorcitinib[tiab] OR "KPL-716"[tiab] OR CIM331[tiab] OR CD14152[tiab] OR "CDX-0159"[tiab] OR CDX0159[tiab] OR "VPD-737"[tiab] OR KHK4083[tiab] OR AMG451[tiab] OR INCB054707[tiab] OR "interleukin-31"[tiab] OR "IL-31"[tiab] OR IL31[tiab] OR "IL-31 receptor"[tiab] OR "IL-31 receptor alpha"[tiab] OR "IL-31 receptor A"[tiab] OR "IL-31RA"[tiab] OR IL31RA[tiab] OR "interleukin-4 receptor"[tiab] OR "IL-4 receptor"[tiab] OR "IL-4R"[tiab] OR "IL-4R alpha"[tiab] OR "interleukin-13"[tiab] OR "IL-13"[tiab] OR "interleukin-5 receptor"[tiab] OR "IL-5 receptor"[tiab] OR "oncostatin M receptor"[tiab] OR "oncostatin M receptor beta"[tiab] OR OSMR[tiab] OR OSMRB[tiab] OR "neurokinin-1"[tiab] OR "neurokinin 1"[tiab] OR "NK1 receptor"[tiab] OR "NK-1 receptor"[tiab] OR "opioid receptor"[tiab] OR "kappa-opioid receptor"[tiab] OR "mu-opioid receptor"[tiab] OR "KIT receptor"[tiab] OR "anti-KIT"[tiab] OR "OX40 receptor"[tiab] OR OX40[tiab] OR biologic*[tiab] OR "monoclonal antibod*"[tiab] OR "targeted therap*"[tiab] OR "targeted treatment*"[tiab] OR "Janus kinase"[tiab] OR "JAK inhibitor*"[tiab] OR "JAK1 inhibitor*"[tiab] OR abrocitinib[tiab] OR upadacitinib[tiab] OR baricitinib[tiab] OR tofacitinib[tiab] OR ruxolitinib[tiab]) AND (randomized controlled trial[pt] OR controlled clinical trial[pt] OR random*[tiab] OR randomly[tiab] OR placebo*[tiab] OR trial*[tiab] OR "phase 2"[tiab] OR "phase II"[tiab] OR "phase 2a"[tiab] OR "phase IIa"[tiab] OR "phase 2b"[tiab] OR "phase IIb"[tiab] OR "phase 3"[tiab] OR "phase III"[tiab]) NOT (animals[mh] NOT humans[mh]) |
| Embase | ('prurigo nodularis':ti,ab,kw OR 'nodular prurigo':ti,ab,kw OR 'chronic nodular prurigo':ti,ab,kw OR 'chronic prurigo':ti,ab,kw OR 'Hyde''s prurigo':ti,ab,kw OR 'prurigo of Hyde':ti,ab,kw OR (prurigo:ti,ab,kw AND nodular*:ti,ab,kw)) AND (dupilumab:ti,ab,kw OR nemolizumab:ti,ab,kw OR vixarelimab:ti,ab,kw OR barzolvolimab:ti,ab,kw OR serlopitant:ti,ab,kw OR nalbuphine:ti,ab,kw OR 'nalbuphine extended-release':ti,ab,kw OR 'nalbuphine ER':ti,ab,kw OR Haduvio:ti,ab,kw OR aprepitant:ti,ab,kw OR tradipitant:ti,ab,kw OR benralizumab:ti,ab,kw OR rocatinlimab:ti,ab,kw OR povorcitinib:ti,ab,kw OR 'KPL-716':ti,ab,kw OR CIM331:ti,ab,kw OR CD14152:ti,ab,kw OR 'CDX-0159':ti,ab,kw OR CDX0159:ti,ab,kw OR 'VPD-737':ti,ab,kw OR KHK4083:ti,ab,kw OR AMG451:ti,ab,kw OR INCB054707:ti,ab,kw OR 'interleukin-31':ti,ab,kw OR 'IL-31':ti,ab,kw OR IL31:ti,ab,kw OR 'IL-31 receptor':ti,ab,kw OR 'IL-31 receptor alpha':ti,ab,kw OR 'IL-31 receptor A':ti,ab,kw OR 'IL-31RA':ti,ab,kw OR IL31RA:ti,ab,kw OR 'interleukin-4 receptor':ti,ab,kw OR 'IL-4 receptor':ti,ab,kw OR 'IL-4R':ti,ab,kw OR 'IL-4R alpha':ti,ab,kw OR 'interleukin-13':ti,ab,kw OR 'IL-13':ti,ab,kw OR 'interleukin-5 receptor':ti,ab,kw OR 'IL-5 receptor':ti,ab,kw OR 'oncostatin M receptor':ti,ab,kw OR 'oncostatin M receptor beta':ti,ab,kw OR OSMR:ti,ab,kw OR OSMRB:ti,ab,kw OR 'neurokinin-1':ti,ab,kw OR 'neurokinin 1':ti,ab,kw OR 'NK1 receptor':ti,ab,kw OR 'NK-1 receptor':ti,ab,kw OR 'opioid receptor':ti,ab,kw OR 'kappa-opioid receptor':ti,ab,kw OR 'mu-opioid receptor':ti,ab,kw OR 'KIT receptor':ti,ab,kw OR 'anti-KIT':ti,ab,kw OR 'OX40 receptor':ti,ab,kw OR OX40:ti,ab,kw OR biologic*:ti,ab,kw OR 'monoclonal antibod*':ti,ab,kw OR 'targeted therap*':ti,ab,kw OR 'targeted treatment*':ti,ab,kw OR 'Janus kinase':ti,ab,kw OR 'JAK inhibitor*':ti,ab,kw OR 'JAK1 inhibitor*':ti,ab,kw OR abrocitinib:ti,ab,kw OR upadacitinib:ti,ab,kw OR baricitinib:ti,ab,kw OR tofacitinib:ti,ab,kw OR ruxolitinib:ti,ab,kw) AND ('randomized controlled trial':ti,ab,kw OR 'controlled clinical trial':ti,ab,kw OR random*:ti,ab,kw OR randomly:ti,ab,kw OR placebo*:ti,ab,kw OR trial*:ti,ab,kw OR 'double blind':ti,ab,kw OR 'single blind':ti,ab,kw OR 'phase 2':ti,ab,kw OR 'phase ii':ti,ab,kw OR 'phase 2a':ti,ab,kw OR 'phase iia':ti,ab,kw OR 'phase 2b':ti,ab,kw OR 'phase iib':ti,ab,kw OR 'phase 3':ti,ab,kw OR 'phase iii':ti,ab,kw) NOT ('animal'/exp NOT 'human'/exp) |
| Cochrane CENTRAL | (("prurigo nodularis" OR "nodular prurigo" OR "chronic nodular prurigo" OR "chronic prurigo" OR "Hyde's prurigo" OR "prurigo of Hyde" OR (prurigo NEAR/3 nodular*)):ti,ab,kw) AND ((dupilumab OR nemolizumab OR vixarelimab OR barzolvolimab OR serlopitant OR nalbuphine OR "nalbuphine extended-release" OR "nalbuphine ER" OR Haduvio OR aprepitant OR tradipitant OR benralizumab OR rocatinlimab OR povorcitinib OR "KPL-716" OR CIM331 OR CD14152 OR "CDX-0159" OR CDX0159 OR "VPD-737" OR KHK4083 OR AMG451 OR INCB054707 OR "interleukin-31" OR "IL-31" OR IL31 OR "IL-31 receptor" OR "IL-31 receptor alpha" OR "IL-31 receptor A" OR "IL-31RA" OR IL31RA OR "interleukin-4 receptor" OR "IL-4 receptor" OR "IL-4R" OR "IL-4R alpha" OR "interleukin-13" OR "IL-13" OR "interleukin-5 receptor" OR "IL-5 receptor" OR "oncostatin M receptor" OR "oncostatin M receptor beta" OR OSMR OR OSMRB OR "neurokinin-1" OR "neurokinin 1" OR "NK1 receptor" OR "NK-1 receptor" OR "opioid receptor" OR "kappa-opioid receptor" OR "mu-opioid receptor" OR "KIT receptor" OR "anti-KIT" OR "OX40 receptor" OR OX40 OR biologic* OR "monoclonal antibod*" OR "targeted therap*" OR "targeted treatment*" OR "Janus kinase" OR "JAK inhibitor*" OR "JAK1 inhibitor*" OR abrocitinib OR upadacitinib OR baricitinib OR tofacitinib OR ruxolitinib):ti,ab,kw) |
| Web of Science | TS=("prurigo nodularis" OR "nodular prurigo" OR "chronic nodular prurigo" OR "chronic prurigo" OR "Hyde's prurigo" OR "prurigo of Hyde" OR (prurigo NEAR/3 nodular*)) AND TS=(dupilumab OR nemolizumab OR vixarelimab OR barzolvolimab OR serlopitant OR nalbuphine OR "nalbuphine extended-release" OR "nalbuphine ER" OR Haduvio OR aprepitant OR tradipitant OR benralizumab OR rocatinlimab OR povorcitinib OR "KPL-716" OR CIM331 OR CD14152 OR "CDX-0159" OR CDX0159 OR "VPD-737" OR KHK4083 OR AMG451 OR INCB054707 OR "interleukin-31" OR "IL-31" OR IL31 OR "IL-31 receptor" OR "IL-31 receptor alpha" OR "IL-31 receptor A" OR "IL-31RA" OR IL31RA OR "interleukin-4 receptor" OR "IL-4 receptor" OR "IL-4R" OR "IL-4R alpha" OR "interleukin-13" OR "IL-13" OR "interleukin-5 receptor" OR "IL-5 receptor" OR "oncostatin M receptor" OR "oncostatin M receptor beta" OR OSMR OR OSMRB OR "neurokinin-1" OR "neurokinin 1" OR "NK1 receptor" OR "NK-1 receptor" OR "opioid receptor" OR "kappa-opioid receptor" OR "mu-opioid receptor" OR "KIT receptor" OR "anti-KIT" OR "OX40 receptor" OR OX40 OR biologic* OR "monoclonal antibod*" OR "targeted therap*" OR "targeted treatment*" OR "Janus kinase" OR "JAK inhibitor*" OR "JAK1 inhibitor*" OR abrocitinib OR upadacitinib OR baricitinib OR tofacitinib OR ruxolitinib) AND TS=(random* OR placebo* OR trial* OR "controlled stud*" OR "double blind*" OR "single blind*" OR "phase 2" OR "phase II" OR "phase 2a" OR "phase IIa" OR "phase 2b" OR "phase IIb" OR "phase 3" OR "phase III") |
| ClinicalTrials.gov | Condition or disease: "Prurigo Nodularis"  Other terms: (dupilumab OR nemolizumab OR vixarelimab OR barzolvolimab OR serlopitant OR nalbuphine OR "nalbuphine extended-release" OR "nalbuphine ER" OR Haduvio OR aprepitant OR tradipitant OR benralizumab OR rocatinlimab OR povorcitinib OR "KPL-716" OR CIM331 OR CD14152 OR "CDX-0159" OR CDX0159 OR "VPD-737" OR KHK4083 OR AMG451 OR INCB054707 OR "interleukin-31" OR "IL-31" OR IL31 OR "IL-31 receptor" OR "IL-31 receptor alpha" OR "IL-31 receptor A" OR "IL-31RA" OR IL31RA OR "interleukin-4 receptor" OR "IL-4 receptor" OR "IL-4R" OR "IL-4R alpha" OR "interleukin-13" OR "IL-13" OR "interleukin-5 receptor" OR "IL-5 receptor" OR "oncostatin M receptor" OR "oncostatin M receptor beta" OR OSMR OR OSMRB OR "neurokinin-1" OR "neurokinin 1" OR "NK1 receptor" OR "NK-1 receptor" OR "opioid receptor" OR "kappa-opioid receptor" OR "mu-opioid receptor" OR "KIT receptor" OR "anti-KIT" OR "OX40 receptor" OR OX40 OR biologic* OR "monoclonal antibod*" OR "targeted therap*" OR "targeted treatment*" OR "Janus kinase" OR "JAK inhibitor*" OR "JAK1 inhibitor*" OR abrocitinib OR upadacitinib OR baricitinib OR tofacitinib OR ruxolitinib)  Study type: Interventional studies  Study status: All statuses |
| Citation tracking | Reference lists of all included randomized controlled trials and relevant systematic reviews were screened. Forward citation tracking was performed for each included report. |

**Supplementary Table S2. Outcome definitions and rules for quantitative synthesis**

| **Domain** | **Original measure/definition** | **Harmonized outcome** | **Follow-up** | **Pooling decision** | **Reason** |
| --- | --- | --- | --- | --- | --- |
| Itch response | WI-NRS or PP-NRS ≥4-point improvement | ≥4-point itch responder | Weeks 4, 8, 12, 16, 24 | Pooled | Clinically comparable scales and threshold |
| Serlopitant itch | Mean itch VAS change | None | Weeks 4 and 8 | Not pooled | Different scale and reporting format |
| Nalbuphine ER itch | ≥30%/≥50% WI-NRS improvement | None | Week 10 | Not pooled | Different responder threshold, timing, and population |
| Sleep | SD-NRS ≥4-point response | Sleep responder | Weeks 4 and 16 | Pooled | Compatible responder definition |
| Other sleep measures | Sleep-loss VAS, MOS-R, difficulty-falling-asleep NRS | None | Variable | Not pooled | Different instruments and reporting formats |

**Supplementary Figure S1. Risk-of-bias judgments by trial**

**Supplementary Figure S2. Summary of risk-of-bias judgments**

**Supplementary Table S3. Pooled secondary efficacy outcomes**

| **Outcome** | **Time point** | **No. of RCTs** | **No. of participants** | **Pooled effect (95% CI)** | **P value** | **I² (%)** |
| --- | --- | --- | --- | --- | --- | --- |
| **Continuous itch-severity outcomes** | | | | | | |
| Percentage change from baseline in weekly average peak/worst itch NRS | Week 4 | 2 | 356 | MD −31.91 (−38.49 to −25.33) | <0.00001 | 0 |
| Percentage change from baseline in weekly average peak/worst itch NRS | Week 8 | 2 | 335 | MD −30.79 (−41.66 to −19.92) | <0.00001 | 28 |
| Percentage change from baseline in weekly average peak/worst itch NRS | Week 12 | 2 | 356 | MD −34.44 (−41.86 to −27.03) | <0.00001 | 0 |
| Percentage change from baseline in weekly average peak/worst itch NRS | Week 24 | 3 | 597 | MD −30.21 (−40.58 to −19.83) | <0.00001 | 67 |
| Absolute change from baseline in weekly average peak/worst itch NRS | Week 8 | 3 | 357 | MD −2.12 (−3.34 to −0.90) | 0.0006 | 56 |
| Absolute change from baseline in weekly average peak/worst itch NRS | Week 12 | 2 | 356 | MD −2.92 (−3.54 to −2.30) | <0.00001 | 0 |
| **Sleep response** | | | | | | |
| SD-NRS ≥4-point response | Week 4 | 2 | 560 | RR 4.42 (2.62 to 7.45) | <0.00001 | 0 |
| SD-NRS ≥4-point response | Week 16 | 3 | 754 | RR 3.04 (2.23 to 4.15) | <0.00001 | 18 |
| **Dermatology-specific quality of life** | | | | | | |
| DLQI change from baseline | Week 16 | 2 | 560 | MD −7.27 (−8.99 to −5.56) | <0.00001 | 32 |
| DLQI change from baseline | Week 24 | 3 | 597 | MD −6.96 (−8.37 to −5.56) | <0.00001 | 27 |
| **Psychological outcomes** | | | | | | |
| HADS-A change from baseline | Week 24 | 3 | 597 | MD −1.51 (−2.11 to −0.92) | <0.00001 | 0 |
| HADS-D change from baseline | Week 24 | 3 | 597 | MD −1.55 (−2.08 to −1.02) | <0.00001 | 0 |

**Supplementary Table S4. Pooled efficacy outcomes by follow-up**

| **Time point** | **WI-NRS/PP-NRS ≥4-point responder** | **Percentage change in itch NRS** | **Absolute change in itch NRS** | **Sleep responder (SD-NRS ≥4)** | **IGA success** | **Dual response** | **DLQI change** | **HADS-A change** | **HADS-D change** |
| --- | --- | --- | --- | --- | --- | --- | --- | --- | --- |
| 4 weeks | ✓ RR 4.76 (3.01–7.55);  I²=5% | ✓ MD −31.91 percentage points (−38.49 to −25.33); I²=0% | — | ✓ RR 4.42 (2.62–7.45); I²=0% | △ RR 3.08 (0.50–18.81); I²=62% | — | — | — | — |
| 8 weeks | △ RR 1.73 (0.94–3.21); I²=0% | ✓ MD −30.79 percentage points (−41.66 to −19.92);  I²=28% | ✓ MD −2.12 points (−3.34 to −0.90); I²=56% | — | ✓ RR 3.38 (1.90–6.01); I²=0% | — | — | — | — |
| 12 weeks | ✓ RR 2.67 (1.43–5.00); I²=61% | ✓ MD −34.44 percentage points (−41.86 to −27.03); I²=0% | ✓ MD −2.92 points (−3.54 to −2.30); I²=0% | — | ✓ RR 3.13 (2.02–4.87); I²=21% | — | — | — | — |
| 16 weeks | ✓ RR 3.32 (2.57–4.29); I²=0% | — | — | ✓ RR 3.04 (2.23–4.15); I²=18% | ✓ RR 3.85 (2.43–6.10); I²=24% | — | ✓ MD −7.27 points (−8.99 to −5.56); I²=32% | — | — |
| 24 weeks | ✓ RR 3.09 (2.18–4.38); I²=0% | ✓ MD −30.21 percentage points (−40.58 to −19.83); I²=67% | — | — | ✓ RR 2.84 (2.04–3.95); I²=0% | ✓ RR 3.98 (2.31–6.85); I²=0% | ✓ MD −6.96 points (−8.37 to −5.56); I²=27% | ✓ MD −1.51 points (−2.11 to −0.92); I²=0% | ✓ MD −1.55 points (−2.08 to −1.02); I²=0% |

**Notes:**

*✓, The 95% CI excludes the null value in the direction favoring intervention.*

*△, The point estimate favors intervention, but the 95% CI crosses the null or is otherwise highly imprecise.*

**Supplementary Table S5. Safety and tolerability outcomes**

| **Safety outcome** | **Studies** | **Participants** | **Pooled effect** | **I²** | **Evidence status** |
| --- | --- | --- | --- | --- | --- |
| Any treatment-emergent adverse event | 11 | 1622 | RR 1.14 (1.06–1.24) | 0% | Clear increase |
| Discontinuation due to adverse events | 10 | 1565 | RR 1.08 (0.61–1.91) | 0% | △ No clear difference |
| Serious adverse events | 11 | 1622 | RR 0.72 (0.48–1.10) | 0% | △ No clear difference |
| Conjunctivitis | 3 | 379 | RR 1.71 (0.53–5.57) | 0% | △ Imprecise |
| Headache | 8 | 1173 | RR 1.37 (0.83–2.25) | 0% | △ Imprecise |
| Edema | 3 | 683 | RR 2.33 (0.73–7.45) | 0% | △ Imprecise |
| Eczematous adverse events | 5 | 961 | RR 2.09 (0.76–5.71) | 15% | △ Imprecise |

**Notes:**

*△, The point estimate favors intervention, but the 95% CI crosses the null or is otherwise highly imprecise.*

**Supplementary Table S6. Sensitivity analyses of outcomes**

| **Outcome** | **Time point** | **Sensitivity analysis** | **Sensitivity measure** | **Sensitivity estimate** | **Sensitivity lower CI** | **Sensitivity upper CI** | **Sensitivity I² (%)** | **Δ I²** | **Statistical significance status unchanged?** |
| --- | --- | --- | --- | --- | --- | --- | --- | --- | --- |
| WI-NRS/PP-NRS ≥4 responder | Week 16 | Restriction to one therapeutic class | RR | 3.41 | 2.46 | 4.73 | 27 | 27 | **Yes** |
| IGA success | Week 8 | Strict outcome-definition restriction | RR | 3.30 | 1.74 | 6.26 | 0 | 0 | **Yes** |
| IGA success | Week 16 | Strict outcome-definition restriction | RR | 4.36 | 2.45 | 7.75 | 37 | 13 | **Yes** |
| SD-NRS ≥4 responder | Week 16 | Exclusion of clinically different trial | RR | 3.18 | 1.83 | 5.55 | 54 | 36 | **Yes** |
| DLQI change | Week 24 | Restriction to replicated drug programme | MD | -6.26 | -7.72 | -4.80 | 0 | -27 | **Yes** |
| Percentage change in itch NRS | Week 4 | Fixed-effect model | MD | -31.91 | -38.49 | -25.33 | 0 | 0 | **Yes** |
| Percentage change in itch NRS | Week 8 | Fixed-effect model | MD | -32.02 | -39.74 | -24.29 | 28 | 0 | **Yes** |
| Percentage change in itch NRS | Week 12 | Fixed-effect model | MD | -34.44 | -41.86 | -27.03 | 0 | 0 | **Yes** |
| Percentage change in itch NRS | Week 24 | Restriction to a replicated drug programme | MD | -24.78 | -32.67 | -16.89 | 0 | -67 | **Yes** |
| Absolute change in itch NRS | Week 8 | Exclusion of a very small early-phase trial | MD | -2.58 | -3.56 | -1.61 | 36 | -20 | **Yes** |
| Absolute change in itch NRS | Week 12 | Fixed-effect model | MD | -2.92 | -3.54 | -2.3 | 0 | 0 | **Yes** |
| HADS-A change | Week 24 | Fixed-effect model | MD | -1.51 | -2.11 | -0.92 | 0 | 0 | **Yes** |
| HADS-D change | Week 24 | Fixed-effect model | MD | -1.55 | -2.08 | -1.02 | 0 | 0 | **Yes** |
| Any TEAE | Final double-blind safety period | Risk-of-bias restriction | RR | 1.15 | 1.05 | 1.27 | 0 | 0 | **Yes** |
| Serious adverse events | Final double-blind safety period | Risk-of-bias restriction | RR | 0.65 | 0.39 | 1.09 | 0 | 0 | **Yes** |
| Adverse events leading to discontinuation | Final double-blind safety period | Risk-of-bias restriction | RR | 1.23 | 0.58 | 2.58 | 0 | 0 | **Yes** |
| Headache | Final double-blind safety period | Risk-of-bias restriction | RR | 1.43 | 0.74 | 2.75 | 0 | 0 | **Yes** |
| Eczematous adverse events | Final double-blind safety period | Risk-of-bias restriction | RR | 2.54 | 1 | 6.42 | 0 | -15 | **No** |
| Eczematous adverse events | Final double-blind safety period | Restriction to the clinically relevant drug class | RR | 2.64 | 0.93 | 7.52 | 4 | -12 | **Yes** |
| Conjunctivitis | Final double-blind safety period | Restriction to the mechanism-relevant drug | RR | 1.98 | 0.31 | 12.79 | 20 | 20 | **Yes** |
| Edema | Final double-blind safety period | Restriction to the mechanism-relevant drug | RR | 1.8 | 0.51 | 6.41 | 0 | 0 | **Yes** |

**Notes:**

*ΔI² was calculated as I² in the sensitivity analysis minus I² in the primary analysis and is expressed in percentage points.*

*Strict-definition: IGA/PN-IGA 0/1 and ≥2-grade improvement*

**Supplementary Table S7. Drug-specific efficacy analyses**

| **Analysis tier** | **Outcome** | **Time point** | **Drug** | **RCTs** | **Participants** | **Effect estimate (95% CI)** | **I² (%)** | **P for interaction** |
| --- | --- | --- | --- | --- | --- | --- | --- | --- |
| **DRUG-STRATIFIED ANALYSES** | | | | | | | | |
| Drug-stratified analysis | Percentage change in peak/worst itch NRS | Week 24 | Dupilumab | 2 | 311 | MD −24.78 pp (−32.67 to −16.89) | 0 | 0.016 |
| Exploratory analyses | Percentage change in peak/worst itch NRS | Week 24 | Nemolizumab | 1 | 286 | MD −39.40 pp (−48.30 to −30.50) | — | 0.016 |
| Drug-stratified analysis | DLQI change | Week 24 | Dupilumab | 2 | 311 | MD −6.26 (−7.72 to −4.80) | 0 | 0.101 |
| Exploratory analyses | DLQI change | Week 24 | Nemolizumab | 1 | 286 | MD −8.40 (−10.50 to −6.30) | — | 0.101 |

**Notes:**

*Between-subgroup comparisons were underpowered and should not be interpreted as evidence of comparative superiority between therapies.*

**Supplementary Table S8. Dose-specific analyses of WI-NRS/PP-NRS ≥4-point response**

| **Analysis tier** | **Outcome** | **Time point** | **Therapeutic class/dose** | **RCTs** | **Participants** | **Effect estimate (95% CI)** | **I² (%)** |
| --- | --- | --- | --- | --- | --- | --- | --- |
| **DOSE-SPECIFIC ANALYSES** |  |  |  |  |  |  |  |
| Dose-selection sensitivity analysis | WI-NRS/PP-NRS ≥4-point response | Week 16 | Nemolizumab trials plus vixarelimab 120 mg every 4 weeks | 4 | 884 | RR 3.12 (2.19–4.44) | 41 |
| Dose-selection sensitivity analysis | WI-NRS/PP-NRS ≥4-point response | Week 16 | Nemolizumab trials plus vixarelimab 360 mg every 4 weeks | 4 | 884 | RR 3.40 (2.63–4.40) | 0 |
| Dose-selection sensitivity analysis | WI-NRS/PP-NRS ≥4-point response | Week 16 | Nemolizumab trials plus vixarelimab 540 mg every 4 weeks | 4 | 884 | RR 3.44 (2.66–4.44) | 0 |

**Supplementary Table S9. Safety outcomes by therapeutic class**

| **Analysis tier** | **Outcome** | **Time point** | **Therapeutic class** | **RCTs** | **Participants** | **Effect estimate (95% CI)** | **I² (%)** | **P for interaction** |
| --- | --- | --- | --- | --- | --- | --- | --- | --- |
| **THERAPEUTIC-CLASS SUBGROUPS** | | | | | | | | |
| Exploratory therapeutic-class subgroup | Any TEAE | Final randomized safety period | IL-31RA blockade (nemolizumab) | 4 | 855 | RR 1.13 (1.01–1.26) | 0 | 0.937 |
| Exploratory therapeutic-class subgroup | Any TEAE | Final randomized safety period | IL-4Rα blockade (dupilumab) | 2 | 309 | RR 1.17 (0.96–1.43) | 0 | 0.937 |
| Exploratory therapeutic-class subgroup | Any TEAE | Final randomized safety period | OSMRβ blockade (vixarelimab) | 2 | 238 | RR 1.16 (0.96–1.39) | 0 | 0.937 |
| Exploratory therapeutic-class subgroup | Serious adverse events | Final randomized safety period | IL-31RA blockade (nemolizumab) | 4 | 855 | RR 0.71 (0.42–1.20) | 0 | 0.813 |
| Exploratory therapeutic-class subgroup | Serious adverse events | Final randomized safety period | IL-4Rα blockade (dupilumab) | 2 | 309 | RR 0.83 (0.25–2.76) | 0 | 0.813 |
| Exploratory therapeutic-class subgroup | Serious adverse events | Final randomized safety period | OSMRβ blockade (vixarelimab) | 2 | 238 | RR 0.45 (0.11–1.96) | 0 | 0.813 |
| Exploratory therapeutic-class subgroup | AE leading to treatment discontinuation | Final randomized safety period | IL-31RA blockade (nemolizumab) | 4 | 855 | RR 1.21 (0.58–2.55) | 0 | 0.333 |
| Exploratory therapeutic-class subgroup | AE leading to treatment discontinuation | Final randomized safety period | IL-4Rα blockade (dupilumab) | 2 | 309 | RR 0.26 (0.03–2.35) | 0 | 0.333 |
| Exploratory therapeutic-class subgroup | AE leading to treatment discontinuation | Final randomized safety period | OSMRβ blockade (vixarelimab) | 2 | 238 | RR 3.11 (0.17–56.65) | 0 | 0.333 |
| Exploratory therapeutic-class subgroup | Headache | Final randomized safety period | IL-31RA blockade (nemolizumab) | 3 | 626 | RR 1.75 (0.75–4.10) | 0 | 0.314 |
| Exploratory therapeutic-class subgroup | Headache | Final randomized safety period | IL-4Rα blockade (dupilumab) | 2 | 309 | RR 0.92 (0.36–2.32) | 0 | 0.314 |
| Descriptive analysis | Headache | Final randomized safety period | OSMRβ blockade (vixarelimab) | 1 | 49 | RR 1.13 (0.25–5.06) | — | 0.314 |
| Mechanism-specific drug-stratified analysis | Eczematous adverse events | Final randomized safety period | IL-31RA blockade (nemolizumab) | 3 | 785 | RR 2.64 (0.93–7.52) | 4 | — |
| Descriptive analysis | Eczematous adverse events | Final randomized safety period | OSMRβ blockade (vixarelimab) | 1 | 49 | RR 2.26 (0.22–23.33) | — | — |
| Descriptive analysis | Eczematous adverse events | Final randomized safety period | NK1R antagonism (serlopitant) | 1 | 127 | RR 0.20 (0.01–4.02) | — | — |
| Mechanism-specific drug-stratified analysis | Conjunctivitis | Final randomized safety period | IL-4Rα blockade (dupilumab) | 2 | 309 | RR 1.98 (0.31–12.79) | 20 | — |
| Mechanism-specific drug-stratified analysis | Edema | Final randomized safety period | IL-31RA blockade (nemolizumab) | 2 | 556 | RR 1.80 (0.51–6.41) | 0 | — |
| Descriptive analysis | Edema | Final randomized safety period | NK1R antagonism (serlopitant) | 1 | 127 | RR 8.86 (0.49–161.27) | — | — |
| Mechanism-specific drug-stratified analysis | New or worsening asthma | Final randomized safety period | IL-31RA blockade (nemolizumab) | 2 | 556 | RR 1.34 (0.36–5.00) | 0 | — |
| Descriptive analysis | New or worsening asthma | Final randomized safety period | OSMRβ blockade (vixarelimab) | 1 | 49 | Not estimable | — | — |
| Descriptive analysis | Death | Final randomized safety period | All active therapies | 11 | 1622 | Not estimable | — | — |

**Supplementary Table S10. GRADE certainty of evidence**

| **Outcome (follow-up)** | **Studies** | **Participants** | **Relative effect (95% CI)** | **Risk of bias** | **Inconsistency** | **Indirectness** | **Imprecision** | **Publication bias** | **Total downgrading** | **Certainty of evidence** |
| --- | --- | --- | --- | --- | --- | --- | --- | --- | --- | --- |
| **CLINICALLY MEANINGFUL ITCH RESPONSE** | | | | | | | | | | |
| PP-NRS ≥4-point response at week 4 | 3 | 630 | RR 4.76 (95% CI 3.01 to 7.55) | Not serious | Not serious | Not serious | Not serious | Undetected | 0 | **HIGH ⨁⨁⨁⨁** |
| WI-NRS ≥4-point response at week 8 | 2 | 72 | RR 1.73 (95% CI 0.94 to 3.21) | Not serious | Not serious | Not serious | Serious | Undetected | 1 | **MODERATE ⨁⨁⨁◯** |
| WI-NRS/PP-NRS ≥4-point response at week 12 | 3 | 381 | RR 2.67 (95% CI 1.43 to 5.00) | Serious | Serious | Not serious | Not serious | Undetected | 2 | **LOW ⨁⨁◯◯** |
| WI-NRS/PP-NRS ≥4-point response at week 16 | 4 | 978 | RR 3.32 (95% CI 2.57 to 4.29) | Not serious | Not serious | Not serious | Not serious | Undetected | 0 | **HIGH ⨁⨁⨁⨁** |
| WI-NRS ≥4-point response at week 24 | 2 | 311 | RR 3.09 (95% CI 2.18 to 4.38) | Serious | Not serious | Not serious | Not serious | Undetected | 1 | **MODERATE ⨁⨁⨁◯** |
| **CONTINUOUS ITCH-SEVERITY OUTCOMES** | | | | | | | | | | |
| Percentage change from baseline in weekly average PP-NRS/WI-NRS at week 4 | 2 | 356 | MD −31.91 percentage points (95% CI −38.49 to −25.33) | Not serious | Not serious | Not serious | Serious | Undetected | 1 | **MODERATE ⨁⨁⨁◯** |
| Percentage change from baseline in weekly average PP-NRS/WI-NRS at week 8 | 2 | 335 | MD −30.79 percentage points (95% CI −41.66 to −19.92) | Not serious | Not serious | Not serious | Serious | Undetected | 1 | **MODERATE ⨁⨁⨁◯** |
| Percentage change from baseline in weekly average PP-NRS/WI-NRS at week 12 | 2 | 356 | MD −34.44 percentage points (95% CI −41.86 to −27.03) | Not serious | Not serious | Not serious | Serious | Undetected | 1 | **MODERATE ⨁⨁⨁◯** |
| Percentage change from baseline in weekly average PP-NRS/WI-NRS at week 24 | 3 | 597 | MD −30.21 percentage points (95% CI −40.58 to −19.83) | Not serious | Serious | Not serious | Not serious | Undetected | 1 | **MODERATE ⨁⨁⨁◯** |
| Absolute change from baseline in weekly average PP-NRS/WI-NRS at week 8 | 3 | 357 | MD −2.12 points (95% CI −3.34 to −0.90) | Not serious | Serious | Not serious | Serious | Undetected | 2 | **LOW ⨁⨁◯◯** |
| Absolute change from baseline in weekly average PP-NRS/WI-NRS at week 12 | 2 | 356 | MD −2.92 points (95% CI −3.54 to −2.30) | Not serious | Not serious | Not serious | Serious | Undetected | 1 | **MODERATE ⨁⨁⨁◯** |
| **SKIN-LESION AND DUAL RESPONSE** | | | | | | | | | | |
| IGA success at week 4 | 2 | 560 | RR 3.08 (95% CI 0.50 to 18.81) | Serious | Serious | Not serious | Very serious | Undetected | 4 | **VERY LOW ⨁◯◯◯** |
| IGA success at week 8 | 4 | 631 | RR 3.38 (95% CI 1.90 to 6.01) | Not serious | Not serious | Not serious | Serious | Undetected | 1 | **MODERATE ⨁⨁⨁◯** |
| IGA success at week 12 | 4 | 871 | RR 3.13 (95% CI 2.02 to 4.87) | Serious | Not serious | Not serious | Not serious | Undetected | 1 | **MODERATE ⨁⨁⨁◯** |
| IGA success at week 16 | 4 | 978 | RR 3.85 (95% CI 2.43 to 6.10) | Not serious | Not serious | Not serious | Not serious | Undetected | 0 | **HIGH ⨁⨁⨁⨁** |
| IGA success at week 24 | 3 | 597 | RR 2.84 (95% CI 2.04 to 3.95) | Not serious | Not serious | Not serious | Not serious | Undetected | 0 | **HIGH ⨁⨁⨁⨁** |
| Dual itch and IGA response at week 24 | 2 | 311 | RR 3.98 (95% CI 2.31 to 6.85) | Serious | Not serious | Not serious | Not serious | Undetected | 1 | **MODERATE ⨁⨁⨁◯** |
| **SLEEP, QUALITY OF LIFE, AND PSYCHOLOGICAL OUTCOMES** | | | | | | | | | | |
| SD-NRS ≥4-point response at week 4 | 2 | 560 | RR 4.42 (95% CI 2.62 to 7.45) | Serious | Not serious | Not serious | Not serious | Undetected | 1 | **MODERATE ⨁⨁⨁◯** |
| SD-NRS ≥4-point response at week 16 | 3 | 754 | RR 3.04 (95% CI 2.23 to 4.15) | Not serious | Not serious | Not serious | Not serious | Undetected | 0 | **HIGH ⨁⨁⨁⨁** |
| DLQI change at week 16 | 2 | 560 | MD −7.27 points (95% CI −8.99 to −5.56) | Not serious | Not serious | Not serious | Not serious | Undetected | 0 | **HIGH ⨁⨁⨁⨁** |
| DLQI change at week 24 | 3 | 597 | MD −6.96 points (95% CI −8.37 to −5.56) | Not serious | Not serious | Not serious | Not serious | Undetected | 0 | **HIGH ⨁⨁⨁⨁** |
| HADS-A change at week 24 | 3 | 597 | MD −1.51 points (95% CI −2.11 to −0.92) | Serious | Not serious | Not serious | Serious | Undetected | 2 | **LOW ⨁⨁◯◯** |
| HADS-D change at week 24 | 3 | 597 | MD −1.55 points (95% CI −2.08 to −1.02) | Serious | Not serious | Not serious | Serious | Undetected | 2 | **LOW ⨁⨁◯◯** |
| **OVERALL AND SERIOUS SAFETY OUTCOMES** | | | | | | | | | | |
| Any treatment-emergent adverse event (final randomized period) | 11 | 1622 | RR 1.14 (95% CI 1.06 to 1.24) | Not serious | Not serious | Serious | Not serious | Undetected | 1 | **MODERATE ⨁⨁⨁◯** |
| Serious adverse events (final randomized period) | 11 | 1622 | RR 0.72 (95% CI 0.48 to 1.10) | Not serious | Not serious | Serious | Serious | Undetected | 2 | **LOW ⨁⨁◯◯** |
| Adverse events leading to treatment discontinuation (final randomized period) | 10 | 1565 | RR 1.08 (95% CI 0.61 to 1.91) | Not serious | Not serious | Serious | Serious | Undetected | 2 | **LOW ⨁⨁◯◯** |
| Death (final randomized period) | 11 | 1622 | Not estimable; one death occurred in a placebo recipient | Not serious | Not serious | Serious | Very serious | Undetected | 3 | **VERY LOW ⨁◯◯◯** |
| **SPECIFIC ADVERSE EVENTS** | | | | | | | | | | |
| Conjunctivitis (final randomized period) | 3 | 379 | RR 1.71 (95% CI 0.53 to 5.57) | Not serious | Not serious | Serious | Very serious | Undetected | 3 | **VERY LOW ⨁◯◯◯** |
| Headache (final randomized period) | 8 | 1173 | RR 1.37 (95% CI 0.83 to 2.25) | Not serious | Not serious | Serious | Serious | Undetected | 2 | **LOW ⨁⨁◯◯** |
| Edema (final randomized period) | 3 | 683 | RR 2.33 (95% CI 0.73 to 7.45) | Not serious | Not serious | Serious | Very serious | Undetected | 3 | **VERY LOW ⨁◯◯◯** |
| Eczematous adverse events (final randomized period) | 5 | 961 | RR 2.09 (95% CI 0.76 to 5.71) | Not serious | Not serious | Serious | Very serious | Undetected | 3 | **VERY LOW ⨁◯◯◯** |

**Notes:**

*RCT evidence started at high certainty. Safety outcomes were downgraded one level for indirectness because cumulative risks were pooled across final randomized treatment periods that varied from approximately 8 to 24 weeks, without person-time standardization.*

*Risk of bias was not downgraded for the safety outcomes because adverse-event ascertainment was generally prespecified and low-overall-risk sensitivity analyses were directionally consistent for the principal safety outcomes.*

*Imprecision judgments considered the 95% CI together with the total number of participants or events and the optimal information size; the number of contributing studies alone was not used as a reason for downgrading. For continuous outcomes with fewer than 400 participants, imprecision was rated serious when a conventional optimal information size was not clearly met, even if the confidence interval excluded no effect. For dichotomous outcomes, imprecision was rated serious when the optimal information size was not met or when the 95% CI crossed no effect and remained compatible with clinically important benefit and harm; it was rated very serious for very sparse events, extremely wide confidence intervals, or non-estimable effects.*

*Percentage change in itch severity at weeks 4, 8, and 12 and absolute change at week 12 were downgraded one level for imprecision because fewer than 400 participants contributed and the conventional optimal information size was not clearly met. Absolute change at week 8 was downgraded for both inconsistency and imprecision.*

*The high-certainty rating for DLQI change at week 16 was retained despite two contributing studies because the total sample size was 560, the 95% CI (−8.99 to −5.56 points) was narrow and excluded no effect, and no serious concerns were identified for risk of bias, inconsistency, or indirectness.*

*Publication bias was not downgraded. Formal assessment was limited by the small number of contributing trials and sparse events; “Undetected” should not be interpreted as evidence that reporting bias is absent.*

*Safety participant counts and pooled effects follow the current manuscript and Supplementary Table S5 and should remain identical across the main text, forest plots, and supplementary materials.*

**Supplementary Figure S3. Funnel plot for any treatment-emergent adverse event**

**Supplementary Figure S4. Funnel plot for serious adverse events**

**Supplementary Figure S5. Funnel plot for discontinuation due to adverse events**
